# Estimating Vehicle Movement Direction from Smartphone Accelerometers Using Deep Neural Networks

**DOI:** 10.3390/s18082624

**Published:** 2018-08-10

**Authors:** Sara Hernández Sánchez, Rubén Fernández Pozo, Luis A. Hernández Gómez

**Affiliations:** Grupo de Aplicaciones de Procesado de Señales (GAPS), Universidad Politécnica de Madrid, 28040 Madrid, Spain

**Keywords:** driving characterization, vehicle movement direction (VMD), accelerometers, Deep Learning, CNN, GRU, t-SNE, PCA

## Abstract

Characterization of driving maneuvers or driving styles through motion sensors has become a field of great interest. Before now, this characterization used to be carried out with signals coming from extra equipment installed inside the vehicle, such as On-Board Diagnostic (OBD) devices or sensors in pedals. Nowadays, with the evolution and scope of smartphones, these have become the devices for recording mobile signals in many driving characterization applications. Normally multiple available sensors are used, such as accelerometers, gyroscopes, magnetometers or the Global Positioning System (GPS). However, using sensors such as GPS increase significantly battery consumption and, additionally, many current phones do not include gyroscopes. Therefore, we propose the characterization of driving style through only the use of smartphone accelerometers. We propose a deep neural network (DNN) architecture that combines convolutional and recurrent networks to estimate the vehicle movement direction (VMD), which is the forward movement directional vector captured in a phone’s coordinates. Once VMD is obtained, multiple applications such as characterizing driving styles or detecting dangerous events can be developed. In the development of the proposed DNN architecture, two different methods are compared. The first one is based on the detection and classification of significant acceleration driving forces, while the second one relies on longitudinal and transversal signals derived from the raw accelerometers. The final success rate of VMD estimation for the best method is of 90.07%.

## 1. Introduction

With the improvement of mobile devices, and especially smartphones, many works related to human activity recognition have been developed using sensor data collected from mobile phones. A particular instance of this area is driving characterization, whose interest resides in a multitude of tasks associated with the driver and safety, such as driver behavior profiling, fuel consumption based on driver characterization, or evaluation of accident blackspots in roads and streets.

Driver behavior characterization covers many areas of study, from applications intended to prevent road accidents, to insurance telematics, where individual policy plans are provided based on “pay as you drive” by rewarding drivers with good driving scores. There are many actions that have effects on traffic safety and that can help to characterize drivers, for instance, hand positioning on the steering wheel, driving speed, staying in the middle of lane or to maintain a space cushion, among others. One of the most influential safety factors is the way to make turns. Some studies, as proposed by Hallac et al. [1], affirm that with the sensor data captured during the same specific turn maneuver, it would be possible to distinguish which driver has done this. Therefore, for drivers’ characterization applications, it could be very useful to identify what type of maneuvers they are carrying out, as well as how they are taking curves, for instance, if a certain event is a turn or not; or, if a more complicated turn event could be divided into more simpler maneuvers, such as acceleration or braking before/within/after the turn. 

Driver characterization methods are usually based on the identification of specific maneuvers, performed by a driver during a journey. In this paper we specifically consider those applications based on smartphone sensor data, where the driver may carry their device in any position inside the vehicle. In these scenarios, before developing any maneuver characterization algorithm, it is crucial to accurately estimate the vehicle movement direction (VMD), which is the “forward” movement direction vector in the smartphone reference system associated with driving along a trip. This direction is characterized by a geometric vector in ${\mathbb{R}}^{3}$ phone’s coordinates, VMD=(X, Y, Z), and is constant along a whole journey (assuming that mobile does not change its position). As stated before, once an accurate estimation of the VMD vector is obtained, several maneuver characterization algorithms can be easily defined. We must point out that the estimation of this principal direction of vehicle movement can be considered equivalent to a calibration process, which can be found in many driving characterization research papers, to map phone coordinates into vehicle reference coordinates. Carvalho et al. [2] and Lu et al. [3] perform this transformation from a smartphone’s coordinate system to a vehicle’s coordinate system. Also, it is mentioned in Kanarachos et al. [4], where this reorientation is carried out by fusing the accelerometer, the gyroscope and the magnetometer signals and calculating the Euler rotation angles, or only with the accelerometer and magnetometer. The main problem with these solutions is that the magnetometer information may be affected by the metal frame of a vehicle, giving us in some circumstances unreliable readings. In our work, VMD estimation relies only on recorded accelerometer data of a smartphone. The main motivation to perform it with only the use of accelerometers, and not use other useful sensors like a gyroscope, is due to a wide range of smartphones on the market that do not include it. In particular, in the middle and low range of smartphones, price matters, so in order to obtain more affordable phones, most models do not include them, using instead virtual gyroscopes that do not offer the same accuracy. A Global Positioning System (GPS) sensor is also not used, thus minimizing significantly the battery consumption.

In this paper we propose a method based on Deep Learning to obtain the VMD vector from accelerometer signals without the need for any other sensors. Once the VMD is obtained it can be used to estimate the longitudinal and transversal acceleration forces of the vehicle and thus to characterize different driving maneuvers. A straight way to obtain the VMD could be to detect a particular maneuver or driving event, such as when the vehicle starts moving forward (Chaudhary et al. [5]). However, this is not a simple task, because when the vehicle starts its movement it does not have to go in a straight direction. Another main problem when classifying directly driving events from temporal sequences, such as accelerometer data, is that they do not have a fixed length (each event has a different duration). Furthermore, it is also difficult to select which are the most representative features to characterize the continuous signal. For these aforementioned reasons, neural networks are becoming more a more popular for event classification tasks using sensors; both to avoid extracting manually patterns or features, and for the good results they have offered, especially when working with large databases. Although neural networks have been shown to be a good way to deal with the problems of driving classification with mobile device sensors, both the way of defining the proper input signals and how to choose the Deep Learning architecture (e.g., how many layers) have a great influence on the final accuracy. Therefore, we will carry out an analysis of different strategies, both for defining inputs and for hyperparameters to the proposed architecture.

In particular, we have designed two different methods to estimate VMD that use a Deep Learning architecture, formed by convolutional and recurrent networks (CNN and RNN). In the first method, we deal with the problem of directly trying to obtain the movement direction vector, through the classification of specific segments of driving events, with different main acceleration forces. In the second method, the VMD estimation is carried out in an innovative way, where the two principal component analysis (PCA) results extracted from acceleration data are taken as inputs to a CNN + RNN architecture trained to predict which one corresponds to the VMD vector.

One of the key points of our work is that the database we use in our experimental setup includes more than 60,000 real driving journeys; both urban and non-urban or mixed, with different durations, recorded in mobile phones with Android as well as iOS operating systems. Our database also includes more than 100 different mobile terminals and more than 3000 different drivers. This provides a unique environment to test relevant results in a complex real-world scenario.

The rest of the paper is organized as follows: work related to driving characterization is summarized in Section 2. In Section 3 a brief introduction to our proposed methodology is given, presenting the two methods developed to obtain the VMD. As both methods employ neural networks, this section is divided into three parts. In Section 3.1, the database of real driving journeys used is presented; Section 3.2 explains the procedure for driving maneuver detection; and Section 3.3 explains how to obtain the ground truth labeling for the neural network, for both methods. Section 4 and Section 5 describe the two methods and results obtained from them. The description of method 1 is divided into four sub-parts: Section 4.1 presents the network architecture; Section 4.2 describes how to evaluate and interpret the neural network model; Section 4.3 presents four different strategies; and Section 4.4 contains a comparison among them. Section 5 develops method 2, explaining how the VMD is obtained from the classification of longitudinal and transversal forces associated with the direction. Section 6 shows a practical example of one of the possible applications of VMD estimation, maneuvers characterization. In Section 7, a comparison with recent works is provided. Finally, in Section 8, we discuss the conclusions and possible future lines.

## 2. Related Work

In this section we review recent works related to driving maneuver detection, using either smartphone sensors or extra equipment (such as On-Board Diagnostic (OBD)). We also examine previous studies that employ frameworks based on Machine or Deep Learning solutions, for activity recognition tasks with motion sensor input signals.

For classifying driving events or maneuvers, previous works use different types of sensors. Castignani et al. [6] suggests combining multiple sensors and GPS information obtained from a smartphone to detect unusual driving events (acceleration, braking and cornering maneuvers), in order to provide a final driving score to drivers. They create an adaptive driver profiling method, which relies on a multivariate normal distribution (MVN), to build a statistical model of a user’s driving characteristics. The proposed system can dynamically build a model for each device and driver, being able to adapt to different environmental conditions. It has also been implemented in both Android and iOS operating systems. However, the use of GPS significantly increases battery consumption.

Meseguer et al. [7] propose the architecture called DrivingStyles, to classify driving styles through the analysis of driver behavior along a journey using an application for Android smartphones. Authors implement a neural network using signals from an OBD-II to characterize the type of road where the vehicle is moving (urban, suburban and highway), and the degree of aggressiveness of each driver. The platform allows Android device users, who have registered in the application, to access to different statistics relating to their driving. However, an OBD-II connector is necessary to send and update the information like acceleration, speed and revolutions per minute to the remote data center, where the neural network is integrated. Ly et al. [8] use the inertial sensor from the controller area network (CAN) bus of the vehicle, to segment and classify driving events in braking, acceleration and turning. Combining turning and braking events, they claim that is possible to recognize the driving style between two similar drivers by means of supervised learning techniques. The work shows that braking and turning events are more useful, compared with acceleration, in order to differentiate between two similar drivers. As future possibilities, they mention being able to differentiate between a greater number of people and to implement it on the smartphone.

Mohan et al. [9] presents a system, called Nericell, in order to monitor road and traffic conditions through a smartphone application. They employ various sensors: the accelerometers to detect potholes/bumps and braking events; the microphone to detect honking; the Global System for Mobile communications (GSM) radio and/or GPS sensors to obtain the localization and also the GPS for the speed. One of the main characteristics of this work is that it faces the problem of monitoring road and traffic conditions in a city, but in developing regions, with a priori more complex and varied patterns. The experiments of the study have been made only for smartphones running in Windows Mobile 5.0, using the same car model, driven by various drivers. On the contrary, Johnson and Trivedi [10] have developed an iPhone application, MIROAD. This uses dynamic time warping (DTW) and smartphone sensors to detect, recognize and record driving maneuvers that are potentially aggressive. Among the sensors used are accelerometers, gyroscopes, magnetometers, GPS and videos. The application allows creating a driving profile for the user. For the tests, several templates were created for each type of event, using a single vehicle and driver. The mobile should be placed in a fixed position, mounted on one arm, ensuring that the camera is not obstructed to capture the video. The processing is in real-time on the smartphone. More works have developed applications for mobile devices; for example Eren et al. [11] propose the use of the accelerometer, gyroscope and magnetometer in an iPhone application to obtain the position, speed, acceleration, deceleration and deflection angle sensory information in order to estimate commuting safety by statistically analyzing driver behavior. This algorithm looks for events like left or right turns, lane departures, acceleration, braking or speed-up. The detection of these events is performed by matching training templates with test data using dynamic time warping and the driving behavior labeling with a Bayesian classifier. Although the experiments have taken place under different weather (rainy, snowy and sunny) and road conditions, only a group of 15 drivers with two experiments for each driver and the same route map have been used. 

In the aforementioned work, Lu et al. [3] present a system for detecting the vehicle mode and the driving activity of travelers. For the vehicle detection, distinguishing between cars, buses or walking among others, they use only accelerometers. However, for driving activity detection, authors use the accelerometer, gyroscope and magnetometer for characterizing driving activities like stopping, going straight, turning left and turning right. Both the classification rates in detecting vehicle modes and in recognizing driving events are high. However, the recognition of activities is limited to the events of four datasets, from three drivers. Also, they propose a method to calculate the optimized data window and also carry out several experiments varying the features and the classification algorithms used.

Works as presented by Ferreira Júnior et al. [12] evaluate the performance of multiple combinations of machine-learning algorithms, such as support vector machines (SVM) or random forest (RF), applied in the motion sensors of Android smartphones to detect aggressive driving events. Data used were recorded only by a smartphone, Motorola XT1058, in a fixed position inside the same car. In total, they had 4 car trips of 13 min each, done by two different drivers. Because they work with a small database, traditional machine learning algorithms are a good solution for driving events detection. However, with larger databases, it can be expected that Deep Learning techniques offer higher recognition results. In fact, in a later work by the same authors (Carvalho et al. [2]) they use the same database to apply Deep Learning and to compare the benefits with different RNN schemes. In their empirical evaluation, the gated recurrent unit (GRU) was the most reliable RNN to be deployed with the accelerometer data; the long short-term memory (LSTM) and the simple RNN have a greater difference depending on the numbers of neurons. Virmani and Gite [13] use video signals as inputs to different Deep Learning configurations for the task of driver assistance, obtaining accuracies from 69% to 95%. The best configuration is formed by a CNN with an LSTM network, recognizing the driver’s behavior while driving, and warning if the action is different to normal activities. The operation is in real-time traffic. Details on what to consider normal actions to drive safely have not been specified. Dong et al. [14] emphasizes the advantages of using Deep Learning instead of using more traditional methods that involve handcrafted features (i.e., feature engineering). For example, with Deep Learning if the driving patterns change in a new data set, the network will be able to adapt to this new circumstance, since the decisions will not be based on the experience in the initial dataset. Sensor data in this solution comprise only GPS and the proposal needs two steps. Firstly, data transformation (from geospatial domain to movement statistics domain), since according to its tests it shows better results than with the raw data; and then, feature learning by the deep architecture. The algorithm proposed by Virmani and Gite [15] is guided to driver behavior identification. Among the different Deep Learning techniques, they have decided to use CNN as good feature extractors, acting on each layer of the network like filters that recognize patterns presented in driving data.

Plötz et al. [16] present the problem of extracting features in a non-systematic way in an activity recognition task with sensors, and how neural networks help in this type of problem (being especially valuable for large data sets). Their tests were performed on accelerometer data only, evaluating the effectiveness of feature learning for activity recognition in four published datasets, and they compare their approach to the literature’s heuristically selected features. Caruana and Niculescu-Mizil [17] do a comparison of several supervised learning algorithms for driving event classification tasks. The supervised algorithms are SVM, neural network, logistic regression, naïve Bayes, memory-based learning, random forest, decision trees, bagged trees, boosted trees and boosted stumps. Also, authors examine the effect of calibrating the models. One of the main conclusions that they extract is that neural networks have the best performance if models are not calibrated after training. One of the significant characteristic of his work is the great variety of performance criteria used to evaluate the learning methods. Other work related to human activity recognition is proposed by Ordoñez and Roggen [18]. They expose how Deep Learning models outperform previous results in OPPORTUNITY data of everyday activity classification and gesture recognition task. Their deep framework is formed by a convolutional network and an LSTM network, each formed by several layers. Among the advantages mentioned are that it is suitable for a multimodal wearable sensor and that they do not require expert knowledge in designing features. Vaizman et al. [19] also use smartphone and smartwatch sensors for automatic recognition of the behavior and environmental context of a person. This classifies work and leisure activities, body movement and modes of transportation, among others. Every minute recorded has multisensor measurements; and, among the main contributions that they mention is that people use the devices naturally and in a free position. Moreover, they named the diversity in smartphones and how the sensor hardware can affect to the measurements. There are also works based on human motion recognition using Deep Learning models, for instance Wang et al. [20], who provide an overview of the most recent advances in RGB-D-based motion recognition. This highlights the success of deep-learning techniques in computer vision, using CNN architectures, widely used in image tasks, and RNN, oriented to sequence problems.

## 3. Our Proposed Work

The main contribution that we propose in this work is the use of Deep Learning techniques as a tool for obtaining a reliable VMD. This movement direction allows the longitudinal and transversal forces associated with the accelerations along a driving journey to be obtained, and then driving maneuvers to be classified reliably or possible dangerous maneuvers detected. In order to obtain this direction, we use only the 3-axis accelerometer from a smartphone, which may be placed in any non-fixed position inside the vehicle.

**Figure 1:**
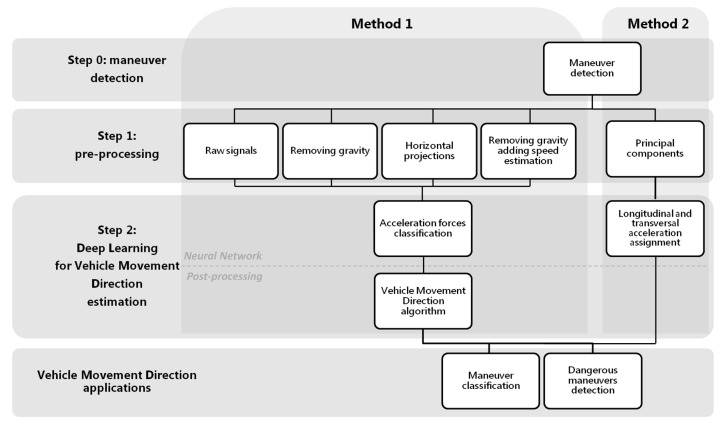
Scheme methods for obtaining vehicle movement direction (VMD).

As mentioned in the previous section, many works focus their studies on characterizing the behavior while driving, but even using more sensors than us they obtain a relatively high number of false alarms of incorrectly classifyied maneuvers. This indicates the complexity of the task. With this purpose of obtaining the VMD by journey, we present two novel methods which will allow us to characterize in depth the driving profile. Figure 1 shows the general scheme of the two proposals that will be developed.

The first step is “Maneuver Detection” (step 0 methods 1&2, Figure 1). The reason for using only zones with possible driving maneuvers as inputs in both methods is that we need areas with enough energy level to capture significant acceleration forces. Three-axis accelerometers collect significant variations of forces in the vehicle; but when these forces stop, the resulting acceleration values captured remain more or less constant. So areas useful for VMD estimation should be zones with enough energy. These areas, from now on called maneuver zones, will be used as inputs to the neural network to classify different tasks. In the first method (Section 4), the Deep Learning architecture will be used to classify the most significant acceleration forces (step 2 method 1, Figure 1) according to some classes; then from the best classified, the VMD will be calculated with a post-processing algorithm. On the other hand, in the second approach (Section 5), the Deep Learning architecture is not used to classify acceleration forces directly; instead, it is used to derive the longitudinal and transversal acceleration forces from the smartphone 3-axis accelerometer signals (step 2 method 2, Figure 1). Step 2 of the block diagram in Figure 1 is simplified, since the network itself will give the VMD. A final part in the scheme has been added, showing possible applications after obtaining the movement direction, applications such as classifying the driving maneuvers or detection of dangerous maneuvers.

### 3.1. Dataset

The dataset used in this study was collected during the deployment of the commercial Drivies App, running on both Android and iOS operating systems (www.drivies.com). Drivies was originally developed by the company Telefónica R&D and recently became a Telefónica spin-off (PhoneDrive S.L., [21]). This application allows the automatic collection of smartphone sensor data from driving journeys without any interaction of a driver that has previously installed Drivies App in her/his smartphone (Drivies users give informed consent to the use of their driving data for the characterization of their driving style). Thanks to collaboration with this company, we had access to 64,397 real driving journeys, of both Android and iOS operating systems, both urban and non-urban, from more than 100 different smartphones and more than 3000 drivers.

For each driving journey our dataset includes signals from the smartphone tri-axial accelerometer and tri-axial gyroscope sensors. Information from gyroscopes has been only used for ground truth labeling of maneuvers and the estimation of VMD. It is important to say that gyroscopes were not used in both methods, neither for the detection of events nor the classification and prediction of the vehicle longitudinal and transversal forces.

### 3.2. Maneuvers Detection

Driving maneuvers detection can be done exclusively with the accelerometer signals obtained from the mobile phone. As mentioned previously, in order to capture the motion sensors of the smartphone it is not necessary to carry the mobile in a fixed position and it can be placed freely.

Maneuvers detection (step 0 methods 1&2, Figure 1) is carried out using the method detailed in [22]. To do this, it is necessary to record accelerometer signals during a driving journey, which are segmented into a number of overlapping data segments (alternatively called windows) of a predefined size. Through a sliding window along the journey, we will decide if that window includes a driving maneuver, such as a turn or acceleration/braking, or a section of the route without event information. To make this decision, we will use the projection of the acceleration on the horizontal plane of driving, in particular the module of said projection. When a minimum of consecutive samples of the projection module exceed a certain energy threshold, we will consider that this segment is susceptible to being a maneuver. Events with excessive energy values will be excluded from this classification, since we will consider them manipulations by the driver or non-controlled phone moves. To decide these thresholds, a study was made on a preliminary database different to the database used in the tests of the present work. We validate the results with the use of the gyroscope and the accelerometers, modifying different experimental thresholds over the horizontal projections of the accelerations, until defining minimum values that detect most of the significant driving maneuvers. Before energy detection, low-pass filtering of the signals is carried out in order to eliminate high-pass frequencies that are not related to driving. For more detail of the process, see [22].

**Figure 2:**
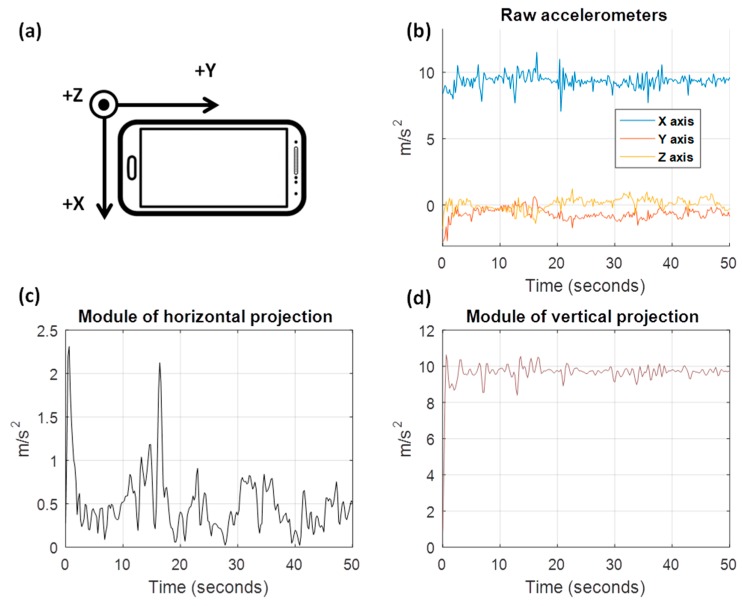
Signals obtained from Android smartphone. (**a**) Position of the mobile while the journey was recorded. (**b**) Raw accelerometers. (**c**) Horizontal projection module. (**d**) Vertical projection module.

One example is shown in Figure 2. Figure 2b displays the raw accelerometers signals captured from an Android terminal. In this, the X axis of the smartphone goes over the width of the screen from left to right, the Y axis over the length of the screen from bottom to top, and the Z axis over the depth of the phone from back to front. Figure 2a shows the possible position in which the driver had the mobile during the journey. In the lower part of the figure, horizontal and vertical projections of the accelerations are drawn.

The module of the horizontal projection is used to remove the effect of gravity on accelerometer signals and to observe exclusively the maneuver’s force. While the vertical projection would pick up the effect of gravity (average value around 9.8 m/s^2^), the horizontal projection will mainly represent driving maneuvers.

After the detection of windows that are likely to be a maneuver, we will perform a more exhaustive centering of the events to improve their location, that is to say, a post-processing to improve the delimitation of the maneuvers. Centering will be carried out by using a background noise threshold. The cutting points of the maneuvers with this threshold will mark the beginning and end of the event. Maneuvers that are very close in time may be fused together as part of a single larger maneuver.

As it is possible to observe in Figure 1, the previous step to the classification, for both method 1 and method 2, is the maneuver detection. Once the beginning and end of the maneuver is detected, these, in turn, will be divided into shorter overlapping windows; so, first maneuver detection and then splitting into overlapping windows. The inputs to the neural network will be these overlapping windows of the maneuvers and in each method a different pre-processing will be carried out. In method 1, the inputs to the neural network architecture will be used to classify the acceleration forces themselves, and in method 2, they will be used to obtain the longitudinal and transversal accelerations associated with the driving. For obtaining results, it will be necessary to create the ground truth for the maneuver classification and estimation of VMD.

### 3.3. Ground Truth Labeling

We will explain how to label the previously detected and segmented maneuvers, which we will use as ground truth for method 1, as well as obtain the longitudinal and transversal signals that will also be used as ground truth for method 2.

***Acceleration Forces Classification for Method 1***

For the classification of the acceleration forces we will use a deep neural network. The main reason to use this is to avoid extracting handcrafted features, an extremely difficult task due to the great diversity of mobile terminals that exist with different behaviors, as well as the great diversity of patterns that it is possible to observe in driving. In addition, signals coming from smartphone sensors are usually very noisy and to find the appropriate distribution in that model is very complicated. Yao et al. [23] discusses why neural networks provide an excellent framework for modeling time-series sensor signals.

In order to obtain our ground truth, we will use accelerometer and gyroscope sensors from mobile phones. Once the database is tagged, we will not use the gyroscopes to classify the maneuvers. The first step will be to detect possible driving maneuvers by the method explained above in Section 3.2. Once they have been detected and segmented, we will classify them in four classes:

- *Braking* (decelerations) without turn, for example when a driver decelerates but stays in the same driving lane.
- *Acceleration* without turn, the same as in the previous class but accelerating, with forces in the opposite direction to the movement.
- Pure *turn*, with transversal forces to the movement.
- All possible combinations of the three above maneuvers, *mixed.* For example, a roundabout, that is to say, braking + turn + acceleration.

This labeling it is necessary for each maneuver, evaluating two criteria: does this maneuver have transversal forces to the movement (i.e., turns)? And longitudinal forces of acceleration or deceleration?

**Figure 3:**
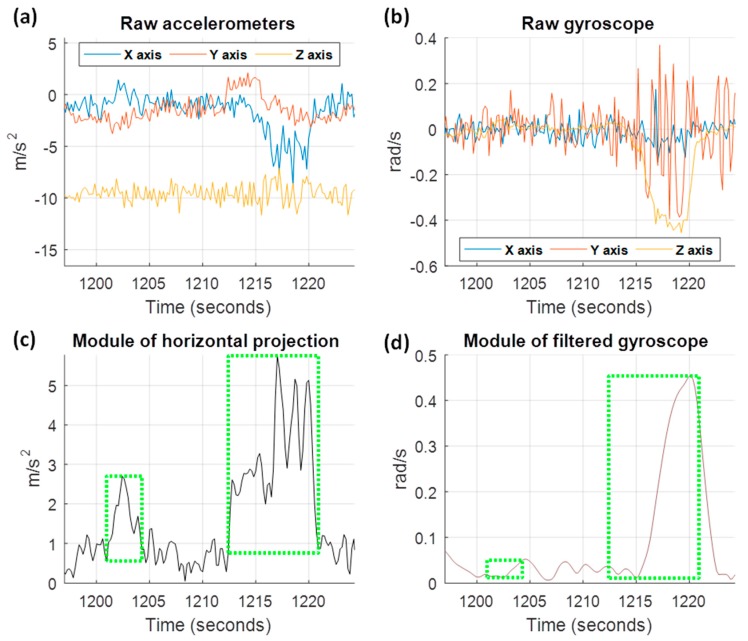
Signals corresponding to two maneuvers detected during a driving journey. (**a**) Raw accelerometers. (**b**) Raw gyroscope. (**c**) Horizontal projection module. (**d**) Module of filtered gyroscope.

To evaluate if the maneuver has transversal and longitudinal forces of acceleration or deceleration, first it is necessary to find out the truth direction of vehicle driving movement, the VMD, in order to be able to compare the maneuvers samples with the driving direction afterwards. To do this, we have to obtain a vector relative to the turns, and two other vectors related to accelerations and decelerations. To obtain a vector relative to the turns, we will use the accelerometer samples of the maneuvers, where the gyroscope module has a value higher than an empirical threshold. The vectors related to the accelerations and decelerations will be obtained with the accelerometer samples of the maneuvers, which do not exceed said threshold. To illustrate this process, Figure 3 shows two real driving maneuvers detected. In the upper part of the plot, the raw signals of the accelerometers and gyroscopes are shown. As the segmentation is made without the help of the gyroscope, exclusively with the accelerometers through the horizontal projection, the delimitation that we obtain for the events are shown with the green boxes in Figure 3c,d. In this example, the samples of the first maneuver will be used to obtain the vectors relative to the accelerations and decelerations (there is not high values of gyroscope); while the samples of the second maneuver will be used for the vector relative to the turns (with high values of the gyroscope).

**Figure 4:**
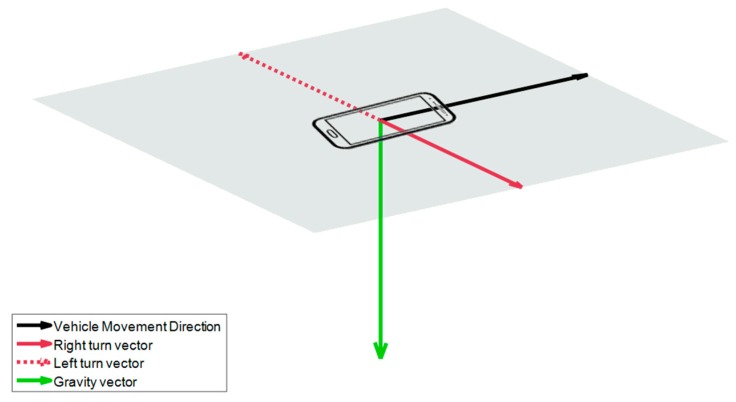
Distribution vectors related to the mobile.

In order to distinguish if a turn is left or right or if we accelerate or decelerate, we will calculate gravity vector estimation in that journey. The vector of the turns must be perpendicular to the vectors of the acceleration and braking, and these vectors must be in a plane perpendicular to the gravity. This will allow us to assign the corresponding sign and therefore obtain the VMD. See Figure 4, where a solid black vector indicates the VMD, which is the same direction as decelerations and opposite to accelerations. This vector and turn direction vectors (divided in right turns, solid red vector, and left turns, dotted red vector) are in a perpendicular plane to the gravity vector, the green vector. 

Once the VMD has been obtained, the maneuvers can be classified in the four mentioned classes by means of the angle between the maneuver samples and this direction; and this process can be performed for each journey.

***Driving Longitudinal and Transversal Acceleration Forces for Method 2***

The process of ground truth extraction for this part is very similar to the previous one, since in order to obtain the longitudinal and transversal signals of the acceleration forces, we also need to estimate previously the VMD vector for each journey. Therefore, we can use the previous driving direction calculated to project the original recorded signals to the longitudinal and transversal directions. To do this, we must multiply the raw accelerometers by the rotation matrices obtained with the longitudinal and transversal vectors directions, obtaining the desired truth signals. 

## 4. Method 1: Vehicle Movement Direction (VMD) Estimation by Acceleration Forces Classification

**Figure 5:**
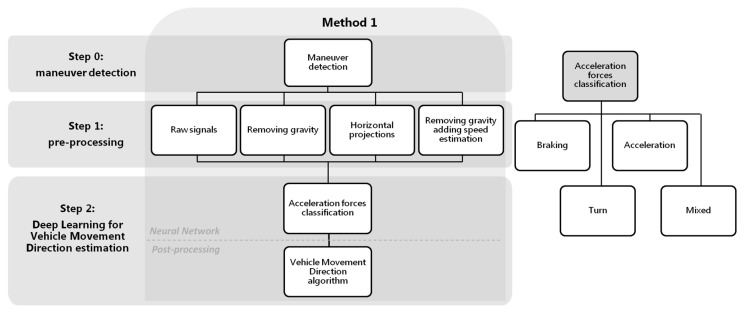
Scheme method 1, maneuvers classification.

We can see in the diagram of Figure 5 the first step, common to the two estimation methods, which is the detection of maneuvers using accelerometers (step 0 method 1, Figure 5). Once the beginning and the end of the specific maneuvers are detected, they will be divided into shorter windows of 10 s with an overlap of 5 s. All the overlapping windows of the maneuvers will be the inputs to the neural network architecture that will be shown later (step 2 method 1, Figure 5), to classify the acceleration forces according to four classes; *braking*, *acceleration*, *turn* and *mixed*. Finally, the most reliable classes will be used in a post-processing algorithm that will allow the VMD to be obtained.

Due to the possible free positions that the driver can carry out the smartphone inside the vehicle, the distribution of the gravity between the tri-axial accelerometers in each journey can be very different, making the model more complex. For it, in this method we analyze different types of pre-processing of the input signals (step 1 method 1, Figure 5), in order to improve the classification performance. The raw accelerometers will be taken as baseline, and we will compare it with the same signals removing the gravity component of the accelerations; with the accelerations projected to a plane perpendicular to the gravity; and, finally, a simple estimation of the speed has also been made, as proposed by Cervantes-Villanueva [24], including this information as one more channel to the neural network. We examine this in more depth later.

### 4.1. Deep Learning Model for Acceleration Classification

**Figure 6:**
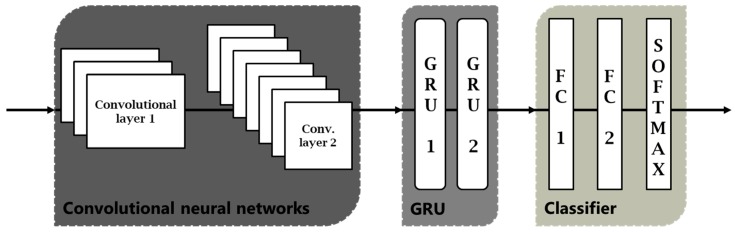
Deep neural network architecture for maneuver classification.

Classification of the driving acceleration forces (step 2 method 1, Figure 5) in *braking*, *acceleration*, *turn* and *mixed* is carried out by means of a network architecture with a CNN of two layers, followed by a RNN of three stacked layers, two fully connected (FC) layers to the output, and finally a Softmax layer, as shown in Figure 6.

The most common interpretation of architecture is that convolutional network layers act as the feature extractor, providing a time-series of feature vectors as input to recurrent networks, that model dynamic information in maneuvers. Ordóñez and Roggen [18], in the area of activity recognition, also use convolutional networks as a feature extractor. As they explain in their work, in domain 1D each kernel can be understood as a filter that helps us to eliminate outliers, applying them on each sensor or each component of the accelerometers in our case. To increase the deeper representation of the data, two convolutional layers have been used. The first layer has 32 filters and the second layer 64 filters; using ReLU as activation function; and performs max pooling, maximum neighborhood, to reduce the dimensionality.

The reason for adding the recurrent networks to the output of the convolutional networks is to be able to learn and model the temporal information, collected in the feature map at the output of the convolutional. The recurrent networks we used were a GRU, because this type offers very good results for time-series modeling. If our input signals will be the accelerometers recorded with the smartphone (or variations of them), this architecture becomes very powerful for learning the sequential features. In addition, the nodes of the network are memory cells, which will allow us to update the states at each time instant, storing the temporal relationships inside the maneuver. Dong et al. [14] compared different architectures and emphasized the benefits of using recurring networks, since these act like unfolded network across time steps. The GRU network consists of three stacked layers, and each GRU layer is formed by 128 neurons.

Since our dataset contains a very large number of maneuvers for training, we expect that the use of stacked layers can significantly improve the results. The output of the last GRU layer is used as input to the last three layers of the architecture, consisting of two fully connected layers and finally a Softmax layer, which will provide us the output probabilities from network scores. The model was trained using an Adam optimizer for minimizing the cross-entropy loss function. Trying to prevent overfitting, a dropout technique was added at the output of the recurrent networks layer; we also used weight decay or L2 regularization; and max pooling in the convolutional layers to reduce the computational cost reducing the number of parameters. Several learning rates were tested, selecting a low value of 0.001, since although the optimization took longer, training results were more reliable than with higher rates. Hyperparameter optimization was done through a grid search using 10-fold cross-validation on the training dataset. The resulting hyperparameters were then used to get the results in the test dataset.

Training phase has been processed on a computer with a Nvidia Tesla K80 graphic card (dual GPU card, with 24 GB of GDDR5 memory, 480 GB/s of memory bandwidth and 4992 CUDA cores). However, the testing phase could be done on a smartphone directly, since it requires a more reasonable consumption of resources. The open source software library TensorFlow^TM^, with Python, was used to build the network.

### 4.2. Model Evaluation and Interpretability

To evaluate the model, different steps of the procedure have been taken into account. Since the purpose of the method is to obtain a reliable VMD, the most important aim will be to compare if the direction estimated is close to the true driving direction, measuring the difference in degrees. In addition, the number of journeys in which it has been possible to estimate VMD will be an important metric (step 2 in method 1 Figure 5 after applying the post-processing algorithm). For method 1, the number of journeys in which VMD can be estimated will depend on the detection or not of maneuvers associated with braking/decelerations or accelerations by the neural networks; that is to say, that neural architecture predicts such classes.

Another mode of evaluation will be the previous step to obtain the VMD in each of the methods presented (step 2 in method 1 Figure 5 before applying the post-processing algorithm). To do this, the different tests will be compared according to the accuracy obtained in the classifications of acceleration forces. Although at the end of the section, a comparative study based on metrics, such as the precision, the recall, the F1 score or the G mean, has been done, the accuracy will be the fundamental metric. It will be specified by the use of confusion matrices obtained for each class.

Not only has the evaluation of the model been considered, but also the interpretation of the training process in the different layers of the Deep Learning architecture, through techniques that allow visualizing data of high dimensionality, like t-distributed stochastic neighbor embedding (t-SNE) (L. van der Maaten and Hinton [25]). This interpretation has been done through the visualization of the projection of the feature vectors obtained in the previous layers to the decision making, that is, in the previous layers to the fully connected layer. In particular, the outputs of the second convolutional network and the output of the recurrent networks GRU of three stacked layers have been analyzed. The objective of visualizing the feature projections, in a function of different parameters, is to observe the influence of these parameters in the decisions that are taken in each layer. For example, to analyze aspects such as whether or not the output information of a layer is independent of the position of the mobile, or whether it begins to show or not a capacity for discrimination of the different types of acceleration forces. t-SNE technique converts the high-dimensional Euclidean distances between data points into conditional probabilities, the similarities. To find the low-dimensional data representation, t-SNE tries to minimize the error between the conditional probability in the high-dimensional data points and in the low-dimensional map points. For the calculation of the similarity between points in the low dimensional space, it employs a Student-t distribution.

When using t-SNE, it is important to consider different values of the perplexity parameter, which roughly represents the number of effective nearest neighbors to consider. Depending on the density of data, some values are usually recommended; generally larger datasets require higher perplexity values. Although the typical range for perplexity is between 5 and 50, depending on the selected value, we can see it reflected in a greater number of clusters. Wattenberg et al. [26] analyzed multiple plots by varying the perplexities. The recommended values by Chaudhary et al. [25] are in a range between 5 and 50, but may change for each data set. For instance, Wattenberg et al. [26] advise that for the t-SNE algorithm to operate properly, the perplexity should have a value smaller than the number of points, and also that in order to avoid weird shapes, the algorithm must be iterated until a stable configuration is reached, since strange figures may be because the process has stopped very soon.

### 4.3. Model Assessment Using Different Pre-Processing Strategies

In this section we present our first results. We have done several tests with different input signals to analyze how this influences classification rates. More specifically, four different approaches have been evaluated:

1. Raw accelerometer signals, the tri-axial component, without any type of pre-processing. These will be used as a baseline.
2. Removing the gravity to each component of the accelerometers, to have the force of the driving maneuvers.
3. Projecting the accelerations to a perpendicular plane to the gravity.
4. Speed estimation added like a channel to the previous approach.

At the end of this section, we present a comparative analysis of these three pre-processing alternatives using several metrics such as accuracy, precision, recall, F1 score or G mean. As well as evaluating the success rates when calculating the VMDs and the number of journeys where this direction can be estimated. Finally, on a smaller dataset, t-SNE projections will be shown to analyze the influence of certain parameters and visualize how it changes for each strategy.

The total journeys have been divided into several subsets; training, validation and testing. For training and validation, k-fold cross-validation was performed, with a k = 10, using a total of 54,403 journeys. Each maneuver is divided into overlapping windows; therefore, the training/validation set has not been divided according to the number of journeys or maneuvers, but as a function of the number of overlapping windows of each class (to avoid skew problems). As we previously mentioned, for this study there are four classes: *braking*; *acceleration*; *turn*; and, *mixed*. A total of 149,157 maneuvers have been used for the training/validation, corresponding to a total of 944,384 overlapping windows already balanced (236,096 of each class), see Table 1 for the dissection by operating system. Finally, different sets of journeys have been used to test and to visualize the classification process in each part. To evaluate the results of the neural network as well as the success rates in obtaining the VMD, a large set of tests has been used, consisting of a total of 9697 journeys of both operating systems (testing 1 in Table 1). Because obtaining the projections of the feature vectors in the different parts of the architecture is very expensive computationally, a smaller test set of 297 examples has been used, with journeys of both operating systems also (testing 2 in Table 1). To test, it has not been necessary to balance the data by class, for this reason the number of overlapping windows by label has not been specified in Table 1 neither for testing 1 nor for testing 2.

#### 4.3.1. Results with Raw Accelerometers

In this first test raw accelerometer signals [X, Y, Z], without any pre-processing, have been used as input to the network. As mentioned, to evaluate the results we will not only take into account if the estimated VMD is close to the true direction (output results of step 2 in method 1 Figure 5 after applying the post-processing algorithm), but also the confusion matrices with the results of neural network classification will be obtained and compared later according to several metrics (output results of step 2 in method 1 Figure 5 before applying the post-processing algorithm).

**Figure 7:**
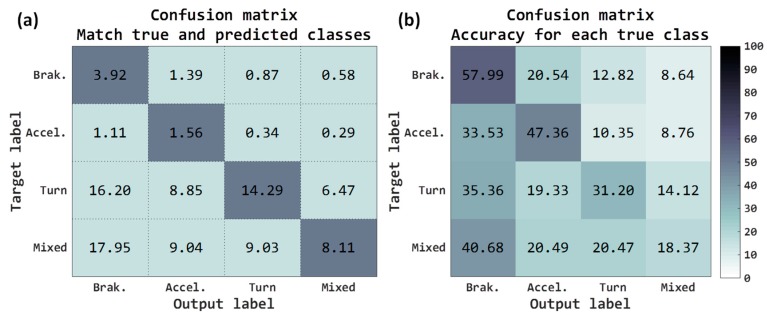
Confusion matrix on raw accelerometers tests: (**a**) according to the number of tested acceleration forces, (**b**) according to the type of force.

The accuracy metric refers to rate of correctly classified acceleration forces windows. Confusion matrices refer to two criteria, the first matrix (see Figure 7a) represents the percentage of acceleration forces windows that have been correctly classified and the second matrix (see Figure 7b) represents the accuracy for each true classes. Both in the first matrix and in the second matrix, the rows are the true class and the columns are the predicted class (output of network).

Total accuracy obtained with the raw accelerometers has been 27.88%, the sum of the main diagonal of the matrix shown in Figure 7a. As mentioned, the test examples have not been balanced, since it is not necessary, and of the 9697 journey, there are some acceleration forces/classes that are more common along the routes. For example, observing the confusion matrix of Figure 7a, the classes more usual are the *turn* classes (45.81%, sum of the third row) and *mixed* classes (44.13%, sum of the fourth row). For instance, in the case of *acceleration*, although 1.56% has been correctly classified, it also shows a high error rate with the *braking* class, 1.11%. If we observe these same results but depending on the accuracy for each true class, Figure 7b, in the case of *acceleration* the right rate is 47.36% and the error rate with the class of *braking* is the 33.53%. The classes with the highest rate have been the *braking* where the 57.99% are correctly predicted as braking.

These preliminary results have revealed that none of the classes worked reliably. In any case, it has to be taken into account that these results are predictions at the level of each window, and the results when calculating the VMD along a whole journey with the post-processing algorithm must be higher, since the algorithm performs a consistency with all window decisions. These raw accelerometer results will be used as the baseline.

Later, these outputs of the neural network are used for the post-processing algorithm applied to each journey in order to obtain the VMD. The details of the algorithm (*Vehicle Movement Direction algorithm*, post-processing step 2 Figure 5) are the following. Firstly, the probabilities obtained in the corresponding overlapping windows will be added together and the final class will be the one with the greater probability after the addition. Only samples that exceed at least 1.5 times the background noise threshold, estimated for the journey, will participate in the calculation of the VMD. With the samples associated to the “good” mixed events (exceeding certain probabilities) a principal component analysis (PCA) will be made to get the two main directions of greater variability. As a result, most of the acceleration and braking samples must fit to one of the previous directions, and the turn samples to other perpendicular direction (also using samples with a certain probability). Thus, the samples of acceleration and braking that do not shift more than 45 degrees from the direction of the principal component associated previously to the longitudinal direction, will be used for the calculation of the VMD. This is as long as they are in sets of at least three consecutive samples, to avoid possible loose samples that deviate the results. Finally, to estimate the final VMD, the average of the resulting acceleration samples without gravity is calculated. To increase the reliability of the results, a higher probability threshold has been required for journeys of less than 5 min, since longer journeys usually have more maneuvers and, therefore, there is more consistency with more decisions along the route, compensating for possible failures of the network.

Below are the results of the VMD obtained, depending on the number of samples that are used for the estimation, see Table 2. A minimum of 15 samples is required, obtaining only a success rate of 50.86% and estimating in 87.79% of the test journeys.

It is possible to observe in the previous table, Table 2, that when demanding more samples to the algorithm of post-processing, the success rate in the directions increases slightly, but reduces the number of journeys where it can be estimated. The results are still very low with only 52.33% of correct directions, demanding 60 or more samples for the calculation, and estimating in 67.67% of test journeys.

#### 4.3.2. Results Removing Gravity from Accelerometer Signals

In this study a pre-processing step is applied to the raw accelerometer signals, before using it as inputs to our Deep Learning architecture. This pre-processing step consists in removing the gravity force from the accelerometer components. For this aim, an estimation of the gravity force has been done, by means of the average of the 3-axis accelerometers in background zones between maneuvers.

**Figure 8:**
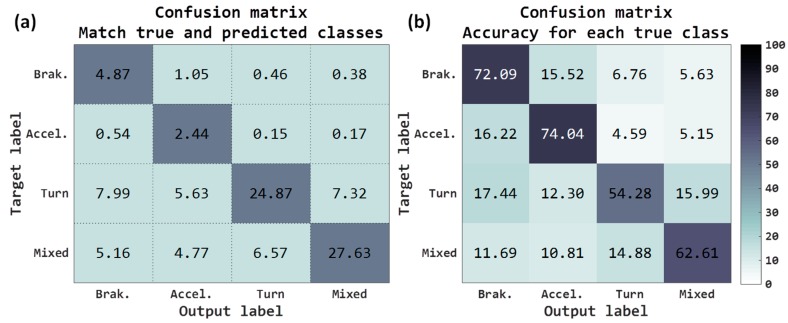
Confusion matrix on raw accelerometers tests removing gravity: (**a**) according to the number of tested maneuvers, (**b**) according to the type of maneuver.

The results at the output of the neural network have been the following: final accuracy obtained for the 4 classes has been 59.81%, a gain of 31.93% with respect to baseline. The classes that have improved the most have been (see Figure 8a) both *brakings* as well as *mixed*. Observing the accuracy for each true class (Figure 8b), all have improved the results, outperforming all of them with 50% success.

Although we really have not normalized the inputs, because of the risk of losing the maneuver information in sensor data, by removing the gravity component of the raw signals we have moved the maneuver values to a more appropriate variation range of input to the neural network. Components that do not carry out gravity information could have a mean near to zero, but components with all gravity information could have a mean around ±9.8 m/s^2^. Removing gravity, we did some kind of “normalization” that could be the cause of this improvement in the classification rates.

The results in the VMD have been (see Table 3) better than those obtained with raw accelerometers; by obtaining better network classifications, the post-processing algorithm results have increased considerably. Demanding 15 samples, a 71.55% of success rate is obtained, and it is possible to calculate this in the 82.86% of the journeys; 20.69% more than in the baseline. Demanding 60 samples, the results go up to 74.86% success, although the routes where this can be calculated is drastically lowered to 51.93%.

#### 4.3.3. Results Using Horizontal Projection of Acceleration forces

Assuming that the driving maneuvers must be in a plane perpendicular to gravity, if we project the accelerations on this horizontal plane, we could observe only the forces related to driving. So using these horizontal projections could improve the classification rates, with respect to the raw accelerometers or the results obtained by removing the gravity from accelerometers. These projections are the same as those used at Section 3.2, in order to delimit the maneuvers limits better.

**Figure 9:**
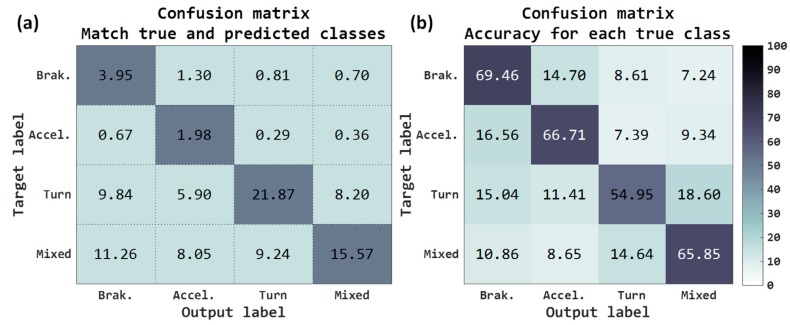
Confusion matrix on horizontal projections tests: (**a**) according to the number of tested maneuvers, (**b**) according to the type of maneuver.

With the components of acceleration projected on the horizontal plane, like input signals to the neural architecture, the total accuracy has been 43.37%, in Figure 9a, which is 15.49% more than that obtained with raw accelerometers. The results are similar in the case of raw accelerometers removing gravity, slightly lower for the case of the most useful classes such as braking and acceleration and slightly higher for the turns and mixed classes, Figure 9b.

Maybe the fact that the deceleration and acceleration classes are slightly lower makes the results of the VMD go down a bit, see Table 4, because the turn class does not help to assign the sign to the VMD properly. Despite the fact that the success rate is lower, the number of journeys where it can be calculated is higher than in the case of raw accelerometers removing gravity, so this solution may be of interest; for example, for 15 samples the rate is 69.76% for the 87.03% of the journeys and in the raw accelerometers removing gravity is 71.55% for the 82.86%. If we increase the samples to 60, the rate is 74.55% for 63.59% of the journeys, while for the other it is 74.86% for the 51.93% of the journeys, that is to say it can be estimated in 11.66% more journeys, failing in 0.31% more.

#### 4.3.4. Results Removing Gravity from Accelerometer Signals and Adding Speed Estimation

The best results have been obtained using as input the raw accelerometers removing the gravity component. In order to try to improve these rates, we have added as additional information the estimation of speed changes analyzing only accelerometer signals. To approximate the speed, we have based this on a technique proposed in Cervantes-Villanueva et al. [24] where speed is estimated in small windows of one second. For each window, the module of the three axes of the accelerometers is calculated at each instant of time and added, multiplying said result by the sum of the time difference between the samples of said window. Once these values have been calculated for each window, the speed at a given time will be the speed at the previous time instant, plus the difference in speeds between the current window and the previous window. Estimating the value of the speed from the three-axis accelerometers is complex and we really only want the information of sudden changes in speed, so we have normalized this value between 0 and 1, and we have introduced this signal as one more channel to the neural network. 

**Figure 10:**
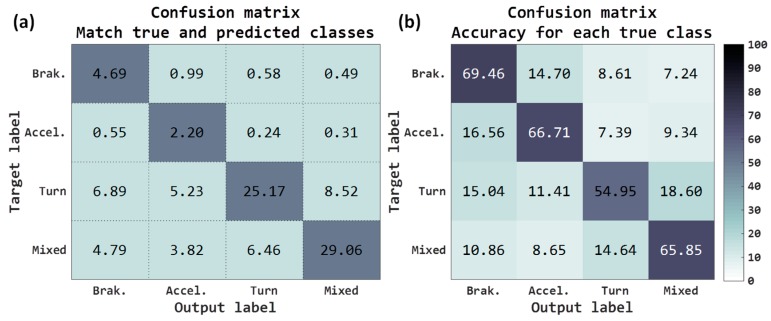
Confusion matrix on raw accelerometers tests removing gravity and adding speed estimation: (**a**) according to the number of tested maneuvers, (**b**) according to the type of maneuver.

Adding this information to the three previous channels, it seems that the classification rates of the network have not improved, see Figure 10, only the mixed class has gone up slightly. So the results when calculating the VMD have not been increased either (see Table 5).

### 4.4. Comparative Study

In this subsection, we will compare both the results obtained from the output of the neural network based on several metrics, as well as the percentage of success in calculating the VMD.

Five different metrics commonly used in the literature have been used: accuracy, which refers to the rate of correctly classified maneuvers; precision, that refers to the proportion of predictions that have been correctly classified; recall, the rate of true labels predicted; F1 score, harmonic mean of precision and recall; and, G mean, geometric mean. The baseline is the classifier with the raw accelerometers as inputs.

**Figure 11:**
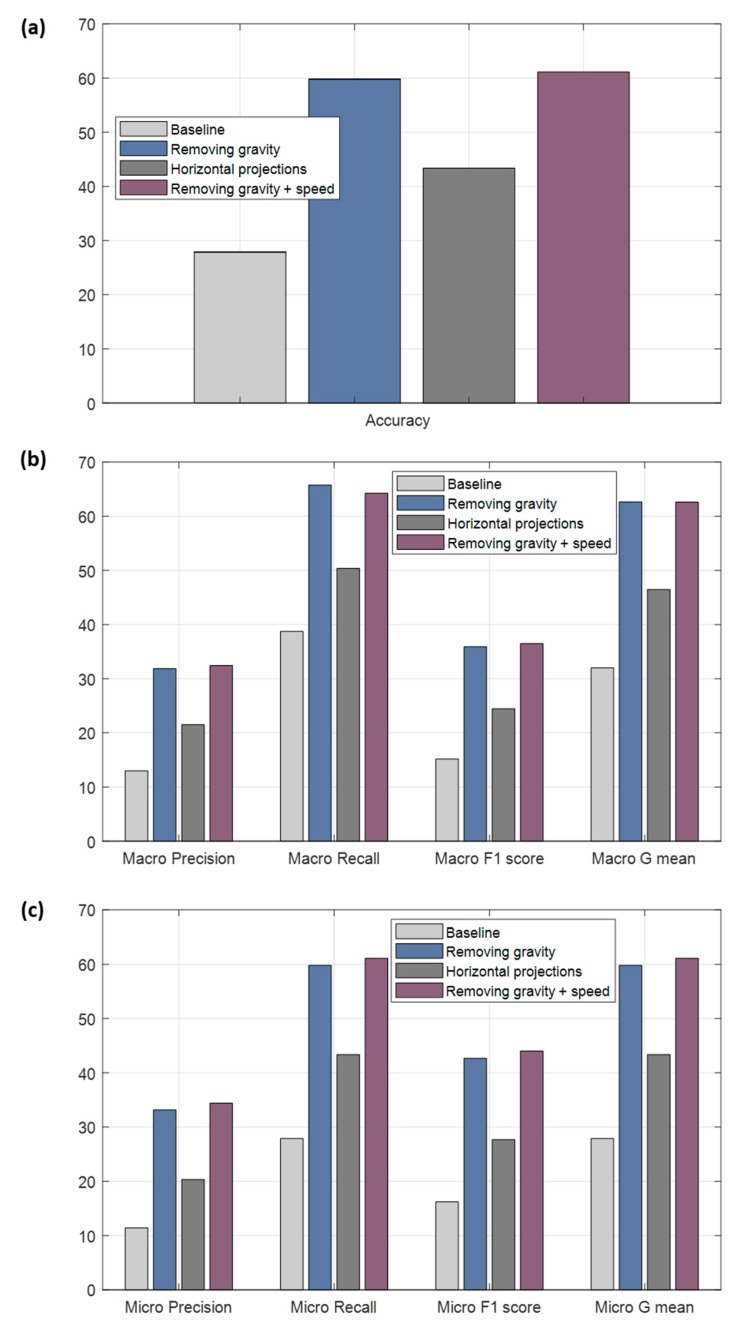
Performance metrics for maneuvers classification. (**a**) Accuracy. (**b**) Macro-averaged metrics. (**c**) Micro-averaged metrics.

As it is a multi-label problem, for each one of the metrics the corresponding formulas have been applied by labels, calculating the metric for each label as if it were a binary classification problem, and then averaging them. To get the mean, two usual forms have been applied: macro-averaged (Figure 11b) and micro averaged (Figure 11c). The main difference is that for macro-averaged, initially the formula of the metric is applied for each class and then these are averaged; and for micro-averaged, firstly it is necessary to add the true positives, false positives, true negatives and false negatives of the six classes and once these four values are obtained, to apply them in the formula of the corresponding metric.

It is possible to observe that the raw accelerometers removing gravity offer the best results compared to other configurations, see Figure 11, going over most of the metrics to the other inputs. The results adding the information of the speed are similar; therefore, we are going to take only the accelerometers without gravity as the best, since they require less processing. The percentage of right maneuvers classified is 59.81% (accuracy), see Figure 11a. The ratio of predictions correct are 31.84% and 33.16% for macro and micro precision respectively; and the accuracy for the true labels are 65.76% for macro recall and 59.81% for micro recall. The F1 score and the G mean are, respectively, 35.89% (macro)/42.67% (micro) and 62.67% (macro)/59.81% (micro). The results of classification are acceptable, since, as mentioned, the task of classifying these four classes is quite complex using only accelerometers. We have improved baseline results but they are still insufficient for the accurate classification and do not provide excessively good results, probably for several reasons. One reason may be the previous nature of the classes, which categorizing them into a pure single class becomes complicated because the classes can easily present patterns of other categories. For example, it is extremely tricky to find a pure turn event (almost always these events have some longitudinal force). It is also important to highlight that until selecting the Deep Learning model used, we tested with different Deep Learning architectures consisting of several convolutional layers plus dense layers at the output, without including the recurrent networks. However, the results obtained were worse more than 5% with respect to the final selected model in the raw accelerometer tests. Among the different recurrent networks tested were the long short-term memory (LSTM) and gated recurrent unit (GRU) networks, but the LSTMs showed a result of around 3% lower average compared to the GRUs.

In the following Table 6 are the results obtained in the estimation of the VMD (percentage of correctly classified journeys) and the number of journeys where it can be calculated, when we require 60 or more samples for each approach. As mentioned, in some cases it may be interesting to obtain the direction in the greatest possible number of journeys or, on the contrary, obtain a higher rate of success despite calculating it in a smaller number of trips. Depending on this criterion, the most appropriate input may vary from projections of accelerations over the horizontal plane or the raw accelerometers without gravity. If we want to increase the reliability, we can demand more samples in the calculation of the VMD, but the percentage of journeys where it is estimated will decrease in all cases.

In order to compare with a baseline system through this new method for estimating VMD based on Deep Learning, we probed other techniques to obtain the VMD based on the idea proposed in Chaudhary et al. [5]. The article says that when the vehicle begins to move, in that initial movement you can obtain the longitudinal acceleration. This idea is actually more complex, because when the vehicle starts the movement it does not have to move in a straight direction, it can go out turning. So we develop a more complicated method that, when an acceleration maneuver or braking maneuver were detected (after and before a stop situation respectively), we calculated the possible VMD. To detect the stops, we trained a neural network that classified the signal as zones of stops or not, in addition to adding a signal-level consistency in the accelerometers. However the success rate achieved was only 62.98% of accuracy, being able to calculate it in a 67.43% of the journeys, a worse result that those obtained with Deep Learning techniques.

Now we are going to try to interpret the training process in the different layers of the Deep Learning architecture. For that, from a smaller set of journeys, the feature vectors obtained in the outputs of the different network layers have been projected, using the t-SNE algorithm. For this purpose, different values of perplexity have been tested, obtaining 50 as the most optimal to visualize the groups. This perplexity value will also be used for the rest of the tests. The first projection corresponds to the feature vector of the test set obtained at the output of the second convolutional layer; the second projection corresponds to the feature vector of the same test set but at the output of the recurrent networks. These feature vectors have been projected in function on the type of class predicted by the network. In order to see the influence of how gravity is distributed among the three axes and the noise threshold of the journey, these projections have also been shown as a function of them.

**Figure 12:**
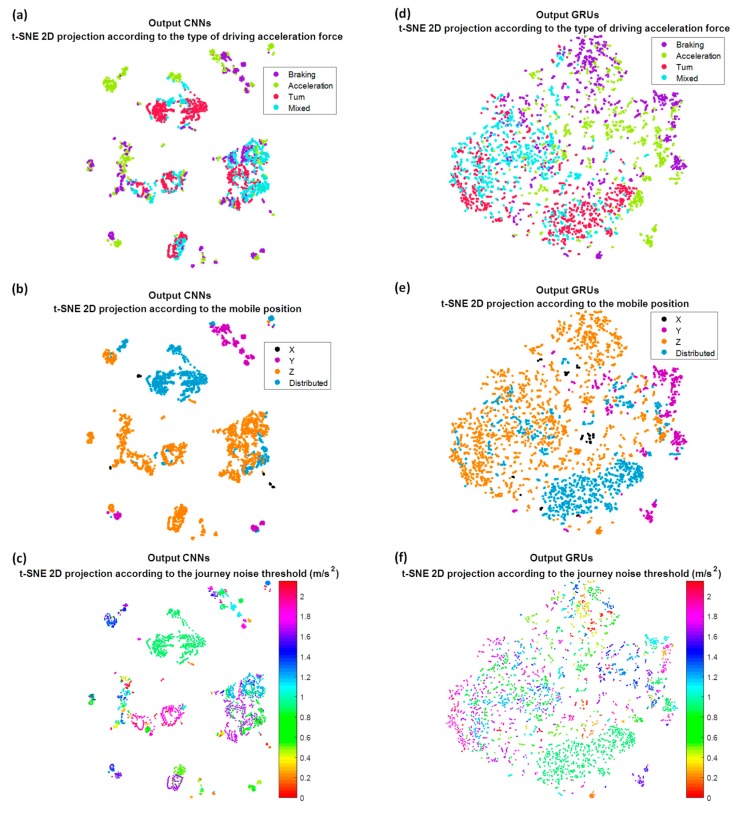
Two-dimensional t-distributed stochastic neighbor embedding (t-SNE 2D) projections of feature vectors for raw accelerometers input signals to the output of the convolutional network (CNN) (left representations) and gated recurrent unit (GRU) network (right representations). (**a**,**d**) Projection depends on type of acceleration force. (**b**,**e**) According to gravity distribution. (**c**,**f**) According to noise threshold of the journey (m/s^2^).

In Figure 12a, we can see these projections when the raw accelerometers are used as input. It is possible to observe how the convolutional network by itself is not able to create four unique clusters associated with the four output categories, although it is already capable of grouping classes of different types that are mixed. On the contrary, the output of the GRU recurrent networks, Figure 12d, seems indeed to distinguish four big clusters, in spite of the results being not optimal. In Figure 12b,e, we draw these same projections but as a function of how gravity is distributed between the axis of the accelerometers. Firstly it is worth emphasizing that the most common mobile position for the drivers is when the Z axis receives a great part of gravity, and the mobile is horizontal with respect to the ground. At the output of the convolutional network, Figure 12b shows that this distribution seems to be very important in network decisions; the X, Y and Z axes appear to be in different groups, except obviously in the case of distributed, when there is no axis that receives most of the gravity. Whereas at the output of the recurrent networks, Figure 12e, although they appear more mixed, it still seems a decisive parameter. Finally, if we represent it as a function of the noise threshold of the journey, something similar to the output of the convolutional network occurs, Figure 12c, but not to the GRU, Figure 12f; it seems that the network is able to discriminate independently of the noise threshold, not perfectly but better than with the gravity distribution.

**Figure 13:**
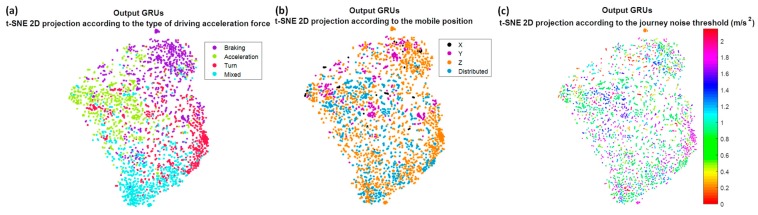
t-SNE 2D projections of feature vectors for raw accelerometers removing gravity input signals to the output of GRU network. (**a**) Projection depends on type of acceleration force. (**b**) According to gravity distribution. (**c**) According to noise threshold of the journey (m/s^2^).

**Figure 14:**
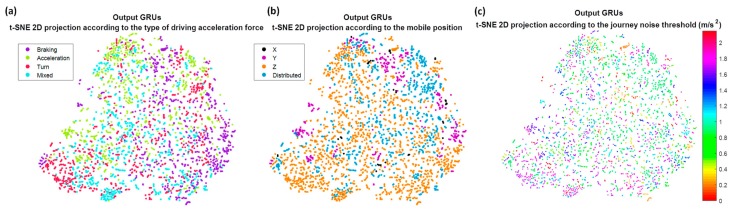
t-SNE 2D projections of feature vectors for horizontal projections input signals to the output of GRU network. (**a**) Projection depends on type of acceleration force. (**b**) According to gravity distribution. (**c**) According to noise threshold of the journey (m/s^2^).

**Figure 15:**
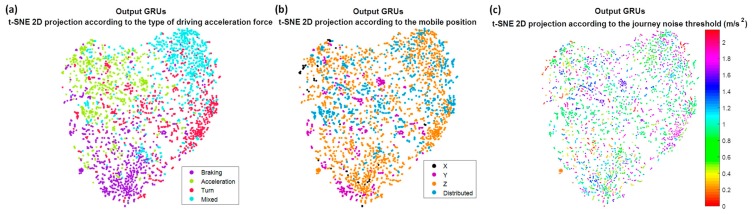
t-SNE 2D projections of feature vectors for raw accelerometers removing gravity input signals plus speed estimation to the output of GRU network. (**a**) Projection depends on type of acceleration force. (**b**) According to gravity distribution. (**c**) According to noise threshold of the journey (m/s^2^).

With the previous results, the importance of the gravity in the decision is highlighted, and this can be one of the reasons why when we eliminate the component of the gravity or we project them to a horizontal plane the rates improve regarding the raw accelerometers. If now we paint these projections of the feature vectors but only at the output of the recurrent networks, for the three remaining strategies: removing gravity (Figure 13), projecting in the horizontal plane (Figure 14) and removing gravity and adding the information of the speed (Figure 15), we can observe the independence with respect to these two parameters, gravity and noise threshold, as well as the input that best groups the clusters into four categories in which we remove the gravity of the raw accelerometers, whether or not we add the speed information.

To summarize this section, this first method has shown its limitations, not being able to estimate the direction in the whole set of routes and obtaining a success rate that does not exceed approximately 75%. Therefore, we propose the following solution, which instead of classifying directly with the acceleration forces, will try to predict the longitudinal and transversal forces.

## 5. Method 2: VMD Estimation by Longitudinal and Transversal Signals Classification 

If the driving longitudinal and transversal forces could be obtained from the raw accelerometers, it would be straightforward to obtain VMD or to classify reliably the maneuvers. The problem is that obtaining these forces from raw accelerometers is not easy and, even using Deep Learning techniques of signal prediction, the results obtained are not very good. So how could we obtain these longitudinal and transversal accelerations for each journey using only the accelerometers?

**Figure 16:**
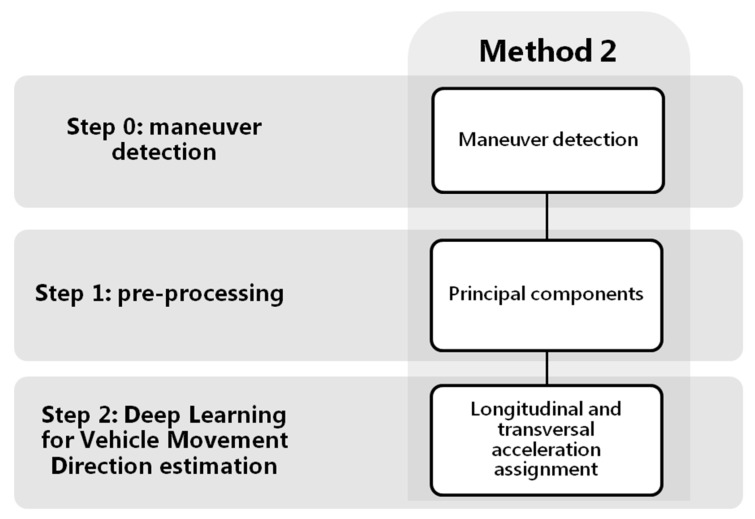
Scheme method 2, driving longitudinal and transversal acceleration forces.

In this work we propose a method to obtain an estimation of the longitudinal and transversal forces associated with the accelerations recorded by the mobile phone. The scheme is shown in the Figure 16. As in method 1, the previous step is the maneuver detection and its later division is in shorter windows of 10 s with an overlapping of 5 s, by means of the segmentation process already described. After obtaining the maneuvers, a pre-processing will be carried out to gain more useful input signals to the neural network. Firstly, we filtered the accelerometer signals to eliminate spurious signals. Secondly, we removed gravity in order to improve the efficiency of the Deep Learning architecture since, when the main components will be calculated, neither the first component nor the second must be gravity. Then we applied a PCA of accelerometer signals only in driving events with the aim of obtaining first and second components with greater variability. The projection on these two first components should be related to driving longitudinal and transversal forces (not necessarily in this order). The third component might be related to forces in gravity plane (i.e., bumps). After calculating the main components, the inputs to the network will be normalized between [−1, 1] to use a similar range of values independent of the component.

**Figure 17:**
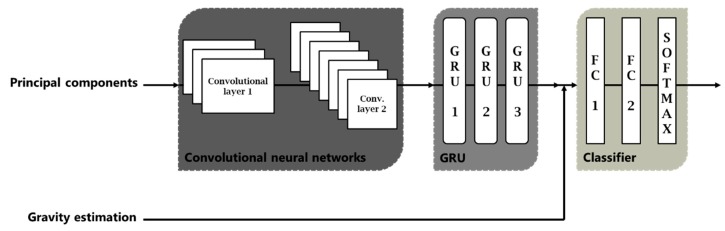
Deep neural network architecture for longitudinal and transversal prediction.

It is not easy to find out which of the two first components of the accelerometers corresponds to the longitudinal and transversal accelerations, since depending on the journey it could be one or another. Therefore, for this correct assignment and estimation of the sign (the sign of PCA component is assigned randomly) we will make use again of Deep Learning techniques, with network architecture as shown in Figure 17. This assignment will be constant along a whole journey. To help in the process of sign assignment to the network architecture, we will add as input the gravity estimation (normalized) of the journey before the first fully connected layer. To obtain ground truth, as in the classification of acceleration forces, we have used smartphones with a gyroscope to be able to deduce the longitudinal and transversal component (*Driving longitudinal and transversal acceleration forces for method 2*, Section 3.3). Then, the gyroscope will not be necessary again.

We want to be able to assign which main component is the longitudinal acceleration and which component is the transversal acceleration and also to determine correctly their sign (this assignment is also maintained along the journey). Therefore, the output of our network will be one of the eight possible combinations that can be produced (see Table 7, PC makes reference to principal component, L and T to longitudinal and transversal respectively). For the training process, the balancing of the data will not be based on the type of maneuver as was done before, but data will be balanced according to the combinations mentioned in Table 7.

**Figure 18:**
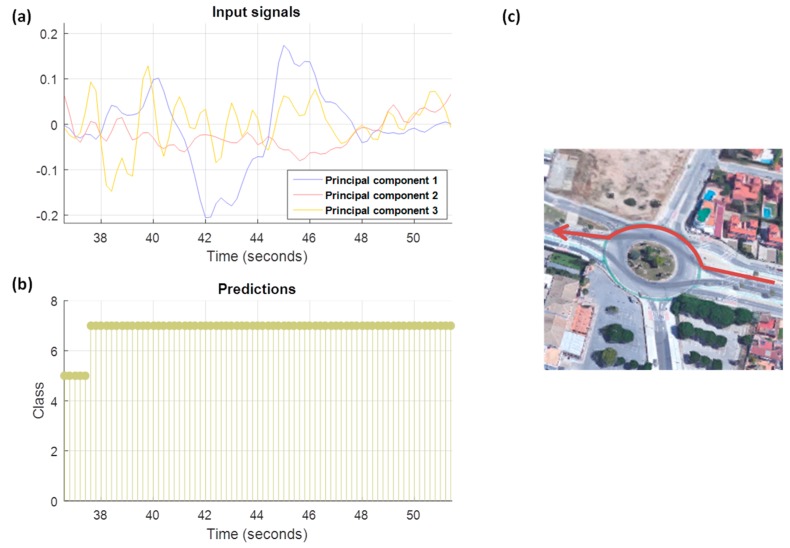
Roundabout maneuver. (**a**) Principal components of the accelerometers. (**b**) Predicted output of the maneuver. (**c**) Maneuver map.

Figure 18 is an example of a driving maneuver. The input signals, Figure 18a, are the projections of the accelerometers on principal components. It is possible to observe in the map obtained through Google Maps (we also have GPS information of journeys), Figure 18c, that the maneuver is a roundabout. Predictions in this method are made with the probabilities of the output of the network in overlapping sliding windows, which are being added at each instant of time for the eight possible combinations. For this particular maneuver, Figure 18b, the correct output is the label 7, where the first principal component represents the transversal component with opposite sign and the second principal component represents the longitudinal component with the same sign. Between the second 36.6 and 37.4, the network architecture has predicted the label 5, and has succeeded in the sign of the longitudinal component, but has failed in the sign of the transversal component. If the decision is taken at the maneuver level, the winning combination is 7.

To make the decisions at journey level, it was decided to add the probabilities of the overlapping windows for all the combinations at each instant of time and finally the results obtained in each time. The final decision will be the one that obtains the highest value. Based on this criterion the results obtained have been the following (Table 8).

Results in Table 8 show that the principal component has been correctly assigned in 93.53% of test journeys to longitudinal or transversal signals. Taking into account not only the assignment, but also the sign of the component, the success rate sign or direction assignment for the longitudinal component is 90.07% and 61.62% for the transversal component. These different accuracy results can be explained considering that driving acceleration and deceleration patterns may differmore from each other than right/left turning patterns. However, we must notice that the transversal sign (right/left) could be easily derived only based on a more accurately detected sign for the longitudinal component. As illustrated in Figure 4, once we know the direction of the longitudinal component, the transversal sign (right/left) component is determined knowing the direction of the gravity force vector. Consequently, beyond the results in Table 8, we can say that the correct assignment of the sign for the transversal component is also 90.07%.

## 6. VMD Application: Accurate Maneuver Classification

Once we have estimated a final VMD, we can use it, for example, to perform a more precise classification of a maneuver as indicated in the diagram of Figure 1. So, we have a vector relative to the longitudinal accelerations that follow the direction of the movement (the VMD), and we also have a vector relative to the transversal accelerations, perpendicular to the movement. Therefore, in order to classify reliably a maneuver, we are going to calculate for each driving event the angle between the VMD vector and the event raw acceleration samples. We considered that belongs to the category of accelerations (accelerations or decelerations/braking) if the average of the all maneuver sample vectors are parallel to the VMD and to the category of turns (left or right turns) if they are perpendicular.

**Figure 19:**
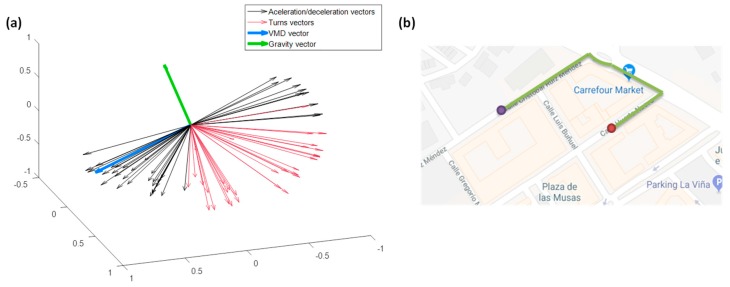
(**a**) Driving maneuver vectors. (**b**) Map maneuver with start at the purple point and end at the red point.

For instance, in Figure 19a, we can see 3-D vectors corresponding to samples of a specific maneuver classified individually according to the type of driving class. In this plot we have the VMD vector in blue, the vector of the gravity in green and the specific maneuver vectors corresponding to the accelerations/decelerations in black and left/right turns in red. It is possible to observe that the vectors of the maneuver are in a plane perpendicular to the gravity, and the acceleration and braking parallel to the direction of the movement, and finally the turns are perpendicular to the movement. If we draw the map of the maneuver (Figure 19b, for this we have used Google Maps), with the beginning of the driving event at the purple point and the end the red point, the succession of most likely actions will be a braking before going through the roundabout, with a right turn at the input and at the output, followed by another section in a straight line with accelerations, as well as another turn to the right again with a slight braking before being followed by an acceleration. This probable succession of actions coincides totally with the results obtained, so it would be possible to apply it in the driving maneuvers to obtain a more precise characterization.

## 7. Comparison with Recent Works

In this section, we evaluate our two developed methods for VMD estimation, making use of the cybernetic theoretical framework (explained in Simpkins and Simpkins [27]). The following Table 9, with the summary results obtained for our proposals, is presented. According to this model, the driver behavior depends on the iterative execution of a repetitive loop of five elements: sensing, information processing, decision making, feedback and action. As in Kanarachos et al. [4] (where they use the cybernetics model to compare, among other things, the transportation mode classification or the aggressive driver behavior), key aspects like the signals, the decision-making framework, the sensor fusion level, the noise rejection, the feedback level and the performance are evaluated. The fusion in both methods is smartphone-based because the raw data do not update to a server that combines signals centrally. In the column of noise is specified No, indicating that there is no probabilistic formulation for rejecting noise and outliers embedded in the signal. The feedback is evaluated only based on its performance, not comparing it with other performance. The metric used for the performance is the accuracy, with 74.86% in method 1 and 90.07% in method 2.

Lu et al. [3] present a comparative table related to recent researches on driving event detection. Although the aim of our work is to calculate the VMD, with this the detection and characterization of driving maneuvers can be also carried out. Most of the works make use of more sensors like the gyroscope; for example Johnson and Trivedi [10] use accelerometer, gyroscope, magnetometer, GPS and video to categorize normal or abnormal driving events and to classify events as left and right turn, U-turns or excessive speed. They place the smartphone in a fixed position for three drivers and three different vehicles, and use dynamic time warping (DTW). The percentage of true positives for Johnson and Trivedi [10] is 91%. Castignani et al. [28] use the accelerometer, the magnetometer, the gravity sensor and the GPS to detect risky driving events (hard acceleration, hard braking, over speeding and aggressive steering) with a fuzzy system using real-time context information (like route topology or weather conditions). In order to measure the accuracy of maneuver detection, they have a single driver performing four different runs with a single car and the smartphone in the car holder. The true positive rate was greater than 90%, requiring a calibration time of at least 17 min and a driven distance of 9.21 km. Other works such as Ferreira Júnior et al. [12] employ different machine learning techniques such as multi-layer perceptron (MLP), SVM, RF or Bayesian networks (BN) to perform driving events characterization. They use the accelerometer, gyroscope, magnetometer and linear acceleration (similar to an accelerometer but excluding the gravity force) in order to detect different driving events like aggressive braking or aggressive acceleration among others. With the smartphone fixed on the car, for four trips of approximately 13 min each on average, the results obtained reach AUC (area under the curve) values between 0.98–0.99. However, they make use of a large number of sensors and also the test dataset is very limited. In fact, they propose as future works to use different vehicles, more Android models, or other driving conditions. Lu et al. [3] present a system that recognizes driving activities like stopping, going straight, turning left or right. They use the accelerometer, the gyroscope and the magnetometer; and they reorient the raw accelerometers from a smartphone’s coordinates into the vehicle’s coordinates. The dataset is formed by three drivers and the mobile position for all activities is in the hand, except for going straight which can also be in the pocket, although in this only two drivers participated in the tests. The result obtained with the use of a RNN is an accuracy of 98.95%. Although the results are good, the database is still limited by the number of different drivers.

One key point of our work, with respect to others that are state of the art, is that we do not need a calibration. In addition, the database is very extensive since more than 60,000 real driving journeys have been used, recorded in mobile phones with Android or OS operating systems, more than 100 different smartphones and more than 3000 different drivers; so we consider the results obtained extremely reliable. Also, significant points are that the device can go in any position for a more natural detection, and that we only use the accelerometers. Using Deep Learning techniques for the best of the methods (second one), an accuracy of 90.07% is obtained in the prediction of the VMD, which ensures the correct classification of the maneuvers in at least that percentage of journeys. Therefore, the results are totally comparable with other studies that use more sensors and thus need more resources.

## 8. Conclusions and Future Work

In this work we propose two novel methods based on Deep Learning for VMD estimation, using only the accelerometer signals captured with smartphones, which can go in any position inside the vehicle.

The first method consists of VMD estimation by means of the classification of different acceleration forces present during a driving journey. The classification of said acceleration forces in classes such as accelerations in the direction opposite to driving or braking in the driving direction, allows using a post-processing algorithm, and obtaining the VMD. Both methods make use of the Deep Learning architecture with two convolutional neural networks, followed by a recurrent neural network of three stacked layers, two fully connected layers and a Softmax layer. However, in method two we do not try to find some acceleration forces, but use the neural network to assign which component of the accelerometers corresponds to the longitudinal acceleration and which to the transversal, with its corresponding signs.

For method one, several tests have been carried out with different input signals in order to classify acceleration forces, because one of the biggest problems when classifying sequences or temporal signals is how to deal with that input signal. Removing the gravity of the raw signals from the accelerometers has improved the results by 31.93% in accuracy, with respect to only the raw accelerometers, probably due to the fact that a certain normalization has been done by removing the gravity to the original maneuvers. Depending on how the gravity is distributed on the axes [X, Y, Z], this causes the average of some or all of the axes to be not above zero, and we can introduce values to the network that present mean ±9.8 m/s^2^ plus the sum of the maneuver force. After obtaining these values, calculating the VMD has also given good results, with more than a 70% success rate. It seems that adding information relative to the speed, speed estimates from the tri-axial accelerometers, does not seem to improve the results, probably because it is difficult to make a reliable estimation without another type of additional information. If instead of using this type of signal we use the projections on the horizontal plane of the accelerations, the results are slightly inferior when we want to classify the acceleration forces, but are good by calculating the VMD since it is able to apply the algorithm in a greater number of journeys, regarding the input of raw accelerometers removing gravity. Using this type of input, we also cause the mean of the input signals to the network architecture to be zero, which can help improve the classification results.

For method two, the input to the network has been the principal component of the accelerometers without gravity and filtered. If we calculate the three main components of the raw accelerometers, the first and the second component must collect the variability associated with driving maneuvers. Therefore, the neural network architecture will be used for said assignment, adding before the first fully connected layer the information of the gravity of that journey. Through this method, it will be attempted to appropriately allocate each main component with the longitudinal and transversal signal. The final accuracy obtained was 90.07%, that is to say, of the total of test trips, 90.07% of the time we are able to know the longitudinal and transversal signal of the associated journey. The advantages with respect to the previous method, in addition to offering better results, is that it can be applied in any journey. Probably one of the reasons why in method 2 the results have improved when calculating the PCA components on input signals is because the principal components are estimated using all the driving maneuvers detected in the whole journey. However, the network does not have this information of whole journey, considering that the input of the net is only a small window of a specific driving maneuver. PCA is an orthogonal linear transformation, which transforms the input vector to a new coordinate system, looking for the greatest variance in each component of the projected space. $A^{\prime }=W\cdot A$, with W the matrix of transformation from original to projected space, $A^{\prime }$ the projected vector and A the original vector. Each whole route has a different projection matrix W, and the network has to learn that information only from a small overlapping window of a specific maneuver window. Those input windows to the network can pick up some variability of the route, but not all, depending on the kind of driving maneuver. Besides that, the network does not have information on when a maneuver belongs to a trip or to a different one. However, when we calculate $A^{\prime }$ using PCA analysis on the whole journey, we will project the input acceleration vector in the directions of maximum variability using the transformation matrix. Through a projection obtained with the information of the whole route, the network should identify that the PCA belongs to the longitudinal or transversal driving component.

Among the possible VMD applications are the maneuvers characterization or the dangerous maneuvers detection. In Section 6 we have shown an example of classification of driving maneuvers.

As future work, other types of input signals could be used for method one, for example in the frequency domain instead of time series. References like [29] propose in their work a method to extract features of the tri-axial accelerometer signals, which are invariant to rotational variations and temporal shift, being independent of the mobile orientation. The method calculates the fast Fourier transform (FFT) of the three accelerometer signals and then the auto-correlation matrix of the complex Fourier features. As a feature vector, they only used the absolute values of the upper triangle components of the auto-correlation matrix including the diagonal. Another future line is to apply the maneuver characterization to the whole database of real journeys available, to make an in-depth study of the type of maneuvers carried out. In addition to classifying the maneuvers according to type, a classification according to different levels of aggressiveness or non-aggressiveness could be carried out analyzing the kind of driver. The characterization of the driver can also be applied to distinguish it; that is to say, for driver identification. Other future work considered is the use of other network architectures, for instance the residual networks, which are deeper than others similarly formed by convolutional networks.

## Figures and Tables

**Table 1:** Number of journeys, maneuvers and overlapping windows used for the acceleration forces characterization, by operating system and dataset.

Dataset	O.S.	Journeys	Maneuvers	Overlapping Windows	Overlapping Windows by Class
Training/Validation	Android	27,526	75,916	507,316	126,829
Training/Validation	iOS	26,877	73,241	437,068	109,267
Testing 1	Android & iOS	9697	162,109	1,864,871	-
Testing 2	Android & iOS	297	3778	40,219	-

**Table 2:** VMD results using raw accelerometers.

Samples Required to Calculate the Final VMD	% of Journeys with Angles between the Estimated and True Directions ≤ 45°	% of Journeys Where It Has Been Calculated
≥15	50.86	87.79
≥30	51.71	80.40
≥45	52.01	73.74
≥60	52.33	67.67

**Table 3:** VMD results using raw accelerometers removing gravity.

Samples Required to Calculate the Final VMD	% of Journeys with Angles between the Estimated and True Directions ≤ 45°	% of Journeys Where It Has Been Calculated
≥15	71.55	82.86
≥30	73.03	71.08
≥45	73.95	60.73
≥60	74.86	51.93

**Table 4:** VMD results using horizontal projections.

Samples Required to Calculate the Final VMD	% of Journeys with Angles between the Estimated and True Directions ≤ 45°	% of Journeys Where It Has Been Calculated
≥15	69.76	87.03
≥30	71.37	78.04
≥45	73.28	71.35
≥60	74.55	63.59

**Table 5:** VMD results using raw accelerometers removing gravity adding speed estimation.

Samples Required to Calculate the Final VMD	% of Journeys with Angles between the Estimated and True Directions ≤ 45°	% of Journeys Where It Has Been Calculated
≥15	67.31	83.03
≥30	68.97	71.15
≥45	69.99	60.67
≥60	70.39	51.23

**Table 6:** Comparison of performances in VMD estimation when we require 60 or more samples.

Samples Required to Calculate the Final VMD	% of Journeys with Angles between the Estimated and True Directions ≤ 45°	% of Journeys Where It Has Been Calculated
Raw accelerometers	52.33	67.67
Removing gravity	74.86	51.93
Horizontal projections	74.55	63.59
Removing gravity & adding speed information	70.39	51.23

**Table 7:** Possible combinations of the input signals.

Combinations	Labels
PC1: L+; PC2: T+	1
PC1: L+; PC2: T−	2
PC1: L−; PC2: T+	3
PC1: L−; PC2: T−	4
PC1: T+; PC2: L+	5
PC1: T+; PC2: L−	6
PC1: T−; PC2: L+	7
PC1: T−; PC2: L−	8

**Table 8:** Results obtained for the longitudinal and transversal component prediction.

Principal Component Assignment	Principal Component and Sign Assignment of the Longitudinal Component	Principal Component and Sign Assignment of the Transversal Component
93.53%	90.07%	61.62%

**Table 9:** Smartphone-based estimation of vehicle movement direction.

VMD Estimation Method	Signals	Framework	Fusion	Noise	Feedback	Performance
Method 1	Accelerometers	Neural Networks	Smartphone based fusion	No	Compared to me	74.86% accuracy
Method 2	Accelerometers	Neural Networks	Smartphone based fusion	No	Compared to me	90.07% accuracy

## References

[1] Hallac D., Sharang A., Stahlmann R., Lamprecht A., Huber M., Roehder M., Leskovec J. Driver Identification Using Automobile Sensor Data from a Single Turn. Proceedings of the 2016 IEEE 19th International Conference on Intelligent Transportation Systems (ITSC).

[2] Carvalho E., Ferreira B.V., Ferreira J., de Souza C., Carvalho H.V., Suhara Y., Pentland A.S., Pessin G. Exploiting the use of recurrent neural networks for driver behavior profiling. Proceedings of the 2017 International Joint Conference on Neural Networks (IJCNN).

[3] Lu D.N., Nguyen D.N., Nguyen T.H., Nguyen H.N. (2018). Vehicle Mode and Driving Activity Detection Based on Analyzing Sensor Data of Smartphones. Sensors.

[4] Kanarachos S., Christopoulos S.R.G., Chroneos A. (2018). Smartphones as an integrated platform for monitoring driver behaviour: The role of sensor fusion and connectivity. Transp. Res. Part C Emerg. Technol..

[5] Chaudhary A., Bajaj A., Shah N., Mankar N. (2013). Mobile Based Monitoring of Driving Patterns. Int. J. Comput. Sci. Manag. Res..

[6] Castignani G., Derrmann T., Frank R., Engel T. Validation Study of Risky Event Classification using Driving Pattern Factors. Proceedings of the 2015 IEEE Symposium on Communications and Vehicular Technology in the Benelux (SCVT).

[7] Meseguer J.E., Calafate C.T., Cano J.C., Manzoni P. DrivingStyles: A smartphone application to assess driver behavior. Proceedings of the 2013 IEEE Symposium on Computers and Communications (ISCC).

[8] Van Ly M., Martin S., Trivedi M.M. Driver Classification and Driving Style Recognition using Inertial Sensors. Proceedings of the 2013 IEEE Intelligent Vehicles Symposium (IV).

[9] Mohan P., Padmanabhan V.N., Ramjee R. Nericell: Rich Monitoring of Road and Traffic Conditions using Mobile Smartphones. Proceedings of the 6th ACM Conference on Embedded Network Sensor Systems.

[10] Johnson D.A., Trivedi M.M. Driving Style Recognition Using a Smartphone as a Sensor Platform. Proceedings of the 2011 14th International IEEE Conference on Intelligent Transportation Systems.

[11] Eren H., Makinist S., Akin E., Yilmaz A. Estimating driving behavior by a smartphone. Proceedings of the 2012 IEEE Intelligent Vehicles Symposium.

[12] Júnior J.F., Carvalho E., Ferreira B.V., de Souza C., Suhara Y., Pentland A., Pessin G. (2017). Driver behavior profiling: An investigation with different smartphone sensors and machine learning. PLoS ONE.

[13] Virmani S., Gite S. Performance of Convolutional Neural Network and Recurrent Neural Network for anticipation of driver’s conduct. Proceedings of the 2017 8th International Conference on Computing, Communication and Networking Technologies (ICCCNT).

[14] Dong W., Li J., Yao R., Li C., Yuan T., Wang L. (2016). Characterizing Driving Styles with Deep Learning. arXiv.

[15] Virmani S., Gite S. (2017). Developing a novel Algorithm for identifying Driver’s behavior in ADAS using Deep Learning. Int. J. Control Theory Appl..

[16] Plötz T., Hammerla N.Y., Olivier P. Feature Learning for Activity Recognition in Ubiquitous Computing. Proceedings of the Twenty-Second International Joint Conference on Artificial Intelligence.

[17] Caruana R., Niculescu-Mizil A. An Empirical Comparison of Supervised Learning Algorithms. Proceedings of the 23rd international conference on Machine Learning.

[18] Ordóñez F.J., Roggen D. (2016). Deep Convolutional and LSTM Recurrent Neural Networks for Multimodal Wearable Activity Recognition. Sensors.

[19] Vaizman Y., Ellis K., Lanckriet G. (2017). Recognizing Detailed Human Context In-the-Wild from Smartphones and Smartwatches. IEEE Pervasive Comput..

[20] Wang P., Li W., Ogunbona P., Wan J., Escalera S. (2018). RGB-D-based Human Motion Recognition with Deep Learning: A Survey. Comput. Vis. Image Underst..

[21] Drivies. https://www.driviesapp.com/.

[22] Pozo R.F., Gomez L.A.H., Meco D.L., Vercher J.B., Muñoz V.M.G. (2014). Method for Detecting Driving Events of a Vehicle Based on a Smartphone.

[23] Yao S., Hu S., Zhao Y., Zhang A., Abdelzaher T. Deepsense: A unified deep learning framework for time-series mobile sensing data processing. Proceedings of the 26th International Conference on World Wide Web.

[24] Cervantes-Villanueva J., Carrillo-Zapata D., Terroso-Saenz F., Valdes-Vela M., Skarmeta A.F. (2016). Vehicle Maneuver Detection with Accelerometer-Based Classification. Sensors.

[25] Maaten L.V.D., Hinton G. (2008). Visualizing Data using t-SNE. J. Mach. Learn. Res..

[26] Wattenberg M., Viégas F., Johnson I. Distill-Latest Articles about Machine Learning. https://distill.pub/.

[27] Simpkins C.A., Simpkins A.M. (2012). Cybernetics: Or Control and Communications in the Animal and the Machine (Wiener, N.) [On the Shelf]. IEEE Robot. Autom. Mag..

[28] Castignani G., Derrmann T., Frank R., Engel T. (2015). Driver Behavior Profiling Using Smartphones: A Low-Cost Platform for Driver Monitoring. IEEE Intell. Transp. Syst. Mag..

[29] Kobayashi T., Hasida K., Otsu N. Rotation invariant feature extraction from 3-D acceleration signals. Proceedings of the 2011 IEEE International Conference on Acoustics, Speech and Signal. Processing (ICASSP).

